# Pediatric glioblastoma cells are sensitive to drugs that inhibit eIF2α dephosphorylation and its phosphomimetic S51D variant

**DOI:** 10.3389/fonc.2022.959133

**Published:** 2022-08-26

**Authors:** Karin Eytan, Ziv Versano, Roni Oren, Jasmine Jacob-Hirsch, Moshe Leitner, Alon Harmelin, Gideon Rechavi, Amos Toren, Shoshana Paglin, Michal Yalon

**Affiliations:** ^1^ Pediatric Hemato-Oncology, Edmond and Lilly Safra Children’s Hospital and Cancer Research Center, Sheba Medical Center, Ramat Gan, Israel; ^2^ Sackler School of Medicine, Tel Aviv University, Tel Aviv, Israel; ^3^ Department of Veterinary Resources, The Weizmann Institute of Science, Rehovot, Israel; ^4^ Sheba Cancer Research Center (SCRC), Chaim Sheba Medical Center, Ramat Gan, Israel; ^5^ Wohl Centre for Translational Medicine, Chaim Sheba Medical Center, Ramat Gan, Israel; ^6^ Chaim Sheba Medical Center, Ramat Gan, Israel

**Keywords:** pediatric glioblastoma, DIPG, eIF2α phosphorylation, PARP-1, raphin-1, salubrinal

## Abstract

We found that pediatric glioblastoma (PED-GBM) cell lines from diffuse intrinsic pontine glioma (DIPG) carrying the H3K27M mutation or from diffuse hemispheric glioma expressing the H3G34R mutation are sensitive to the combination of vorinostat (a histone deacetylase inhibitor) and PARP-1 inhibitors. The combined treatment increased the phosphorylation of eIF2α (P-eIF2α) relative to each drug alone and enhanced the decrease in cell survival. To explore the role played by increased P-eIF2α in modulating PED-GBM survival and response to treatments, we employed brain-penetrating inhibitors of P-eIF2α dephosphorylation: salubrinal and raphin-1. These drugs increased P-eIF2α, DNA damage, and cell death, similarly affecting the sensitivity of DIPG cells and derived neurospheres to PARP-1 inhibitors. Interestingly, these drugs also decreased the level of eIF2Bϵ (the catalytic subunit of eIF2B) and increased its phosphorylation, thereby enhancing the effect of increased P-eIF2α. Transient transfection with the S51D phosphomimetic eIF2α variant recapitulated the effect of salubrinal and raphin-1 on PED-GBM survival and sensitivity to PARP-1 inhibitors. Importantly, either salubrinal or raphin-1 dramatically increased the sensitivity of DIPG cells to radiation, the main treatment modality of PED-GBM. Finally, PED-GBM was more sensitive than normal human astrocytes to salubrinal, raphin-1, and the treatment combinations described herein. Our results indicate that combinations of histone deacetylase inhibitors and PARP-1 inhibitors should be evaluated for their toxicity and efficacy in PED-GBM patients and point to drugs that increase P-eIF2α or modulate its downstream effectors as a novel means of treating PED-GBM.

## Introduction

Pediatric glioblastomas (PED-GBM) harboring somatic heterozygous missense H3K27M or H3KG34R/V/D mutations are invariably fatal. H3-mutant PED-GBM are distinguished by chromatin modifications, which lead to altered gene expression supporting their specific genetic programs (1–7). H3K27M mutation characterizes the diffuse midline GBM (DMG) tumors located in the pons (DIPG), cerebellum, thalamus, and spine, while H3G34R/V/D-mutant GBM tumors are found in the hemispheric region (8). Consequently, a significant percentage of these tumors are either completely or partially inaccessible for surgical resection. To date, the main treatment modality of PED-GBM is ionizing radiation, which provides only temporary relief. In addition, unlike in adult GBM, the addition of temozolomide in PED-GBM did not show a survival benefit compared to radiation therapy alone, even in tumors with methylated O^6^methylguanine-DNA-methyltransferase (MGMT) promoter (9).

Grasso et al. demonstrated the inhibitory effect of panobinostat, a pan-histone deacetylase inhibitor (HDACi), on the growth of DMG human cell lines in culture and in tumor xenograft models (10), while Chornenkyy et al. showed that niraparib, the brain-penetrating PARP-1 inhibitor (PARPi), decreased survival and enhanced the sensitivity of pediatric high-grade astrocytomas to ionizing radiation both in cell culture and in a tumor xenograft model (11).

Relevant to these studies are the findings that HDACis sensitize breast, ovarian, myeloid leukemia, and prostate cancer cells to treatment with PARPis, regardless of their innate capacity for repairing dsDNA breaks (12–15). We have also previously shown that the combination of vorinostat and veliparib (an HDACi and a PARPi, respectively) enhanced the killing of breast cancer and GBM cell lines, including H3.3G34V-expressing KNS-42 cells (12, 16, 17). To the best of our knowledge, the effect of combining HDACis and PARPis on DMG survival has not been reported to date in either preclinical or clinical trials.

In search of vorinostat downstream effectors that mediate its sensitizing effect on the cellular response to PARPis, we found that vorinostat increased the level of phosphorylation of eIF2α (P-eIF2α) in PARPi-treated cells twofold relative to PARPi treatment alone, a phenomenon that in and of itself was sufficient to increase the cellular sensitivity to PARPis (12, 16).

eIF2α is a subunit of eIF2, which forms a ternary complex with GTP and tRNA^iMET^. This complex transfers tRNA^iMET^ to the small ribosomal subunit, which, in association with other initiation factors, scans the mRNA until it reaches the start codon, where it is joined by the large ribosomal subunit. Upon recognition of the start codon, GTP is hydrolyzed, and eIF2·GDP is released. Replenishment of eIF2·GTP is executed by eIF2B, a decameric GDP/GTP exchange factor. However, during cellular stress, eIF2α is phosphorylated, turning eIF2 from a substrate of eIF2B into its inhibitor, leading to decreased levels of the ternary complex and, with it, to a global attenuation of protein translation (18). In addition to the interaction with P-eIF2α, the activity of eIF2B is also regulated by the phosphorylation of its catalytic epsilon subunit. Decreased phosphorylation of eIF2Bϵ at S539 increases the GDP/GTP exchange activity of eIF2B and diminishes the inhibitory effect of P-eIF2α (19, 20). Due to its relatively low cellular level, eIF2B is a rate-limiting factor in the eIF2·GDP/GTP exchange reaction (18). Thus, even relatively small changes in the cellular level of P-eIF2α, phosphorylated eIF2Bε (P-eIF2Bε) or eIF2Bε may have an impact on the rate of protein translation.

The global attenuation of protein translation goes hand in hand with the increased translation of a subset of mRNAs that mostly code for proteins that modulate the protective response to stress, such as transporters and transcription factors (18, 21). However, to allow for the translation of the newly transcribed mRNAs, the increased P-eIF2α has to be transient (22). Therefore, a sustained and excessive elevation in P-eIF2α often leads to cell death (23, 24).

Dephosphorylation of P-eIF2α is accomplished by protein phosphatase 1 (PP1) in complex with either one of its two regulatory subunits—the constitutive CReP or the stress-induced GADD34—and with G-actin (25–27). The physiological outcome of increased P-eIF2α has been assessed by employing inhibitors of eIF2α dephosphorylation (salubrinal and raphin-1) and the non-phosphorylatable (S51A) and phosphomimetic (S51D) eIF2α variants (16, 28–30). Salubrinal inhibits eIF2α dephosphorylation by interfering with the interaction of PP1 with its regulatory subunits CReP and GADD34 (28). Its inhibitory effect on isolated hollophosphatase is reproducible, although milder than that observed within the cells (31). At relatively low concentrations, raphin-1 inhibits the recruitment of P-eIF2α to the PP1-CReP complex, while at concentrations higher than 10 µM, it also inhibits the activity of PP1 when in complex with GADD34. Its inhibitory effect on the activity of isolated hollophosphatase is similar to that observed within cells (32). Both salubrinal and raphin-1 penetrate the blood–brain barrier, but unlike salubrinal, raphin-1 did not adversely affect the long-term memory of the mice (32).

Another compound that can assist in evaluating the role of a moderate increase in cellular P-eIF2α is ISRIB. Although ISRIB and P-eIF2α bind to eIF2B at different sites, they do so in a mutually exclusive manner. Therefore, at low P-eIF2α levels, ISRIB binds to and stabilizes eIF2B, thereby counteracting the effect of increased P-eIF2α. However, when P-eIF2α is sustained and excessive, ISRIB fails to bind to and stabilize eIF2B (33).

Modulation of P-eIF2α also affects the response of cells to anti-neoplastic treatments. While mouse embryonic fibroblasts expressing S51A-eIF2α are more sensitive to doxorubicin and HDACis than their wild-type counterparts, increased P-eIF2α mediates IL24-induced death of squamous cell carcinoma, an effect diminished upon the expression of S51A-eIF2α (34, 35). Also, ONC201 which demonstrated clinical efficacy toward DMG patients (36) is known to decrease survival and increase P-eIF2α in colon carcinoma cell lines (37). In addition, we have previously demonstrated that a sustained increase in P-eIF2α mediates the anti-neoplastic activity of ionizing radiation and vorinostat in a variety of human breast cancer cell lines (16).

In the experiments reported herein, we evaluated the effect of increased P-eIF2α on PED-GBM survival and response to treatments. Toward that end, we employed raphin-1, salubrinal, and plasmids expressing eIF2α phosphomimetic variants. The results indicated that increased P-eIF2α decreases PED-GBM survival and increases PED-GBM’s sensitivity to PARPis and ionizing radiation. Increased sensitivity to ionizing radiation is of particular importance in light of the resistance of DIPG to this sole available treatment and the absence of a clinically effective radio enhancer. In addition, to the best of our knowledge, this report shows for the first time the direct effect of increased P-eIF2α on DNA integrity in cancer cells and the effect of raphin-1 and salubrinal on the ratio of P-eIF2Bϵ to eIF2Bϵ (P-eIF2Bϵ/eIF2Bϵ). The possible mechanisms underlying these two phenomena are discussed herein.

## Materials and methods

### Cell lines

SU-DIPG-VI-GFP-LUC (SU-DIPG-VI) and SU-DIPG-IV cell lines derived from pediatric DIPG primary tumors expressing H3.3K27M and H3.1K27M, respectively, were a generous gift from Dr. Michelle Monje (Stanford University, Stanford, CA). The KNS-42 cell line, which was derived from a 16-year-old patient glioma and harbors H3.3G34V mutation, was obtained in 2019 from the Japanese Collection of Research Bioresources Cell Bank (Osaka, Japan). Following a short propagation period, these cell lines were frozen, and aliquots were resuscitated and used for 8 weeks. Normal human astrocytes (HAs) were obtained from ScienCell Research Laboratories (Carlsbad, CA). All the cells were routinely tested for the presence of mycoplasma.

### Growth conditions

KNS-42 cells were grown in Eagle’s minimal essential medium, supplemented with 5% fetal bovine serum (FBS), 1% L-glutamine, penicillin, and streptomycin (Biological Industries, Kibbutz Beit-Haemek, Israel). SU-DIPG-VI and SU-DIPG-IV cells were grown in a tumor stem medium consisting of 50% Neurobasal™-A Medium, 50% DMEM/F-12, 1% HEPES (1 M), 1% sodium pyruvate, 1% non-essential amino acids, 1% GlutaMAX and 1% antibiotic antimycotic, 2% B-27 (Thermo Fisher Scientific, Waltham, MA), 0.02% heparin (STEMCELL Technologies, Canada), 20 ng/ml EGF and bFGF, and 10 ng/ml PDGF-AA and PDGF-BB (PeproTech Asia, Rehovot, Israel). HA cells were grown in complete astrocyte medium supplemented with 1% penicillin and streptomycin, 2% FBS, and 1% astrocyte growth supplements (all from ScienCell).

### Reagents

Vorinostat was obtained from LC Laboratories (Boston, MA), olaparib and niraparib were obtained from MedChemExpress (Monmouth Junction, NJ), salubrinal was obtained from Sigma-Aldrich (Merck KGaA, Darmstadt, Germany), raphin-1 was obtained from Tocris Bioscience (Bristol, UK), and veliparib was obtained from APExBIO (Houston, TX). All the drugs were added from stock solutions in dimethyl sulfoxide (DMSO), and the control cultures received equal amounts of the vehicle. The final concentration of DMSO in the culture medium did not exceed 0.1%. All the other materials were of analytical grade.

### Survival assays

SU-DIPG-VI cells were plated in triplicates at a density of 3,500 cells per well in 96-well plates (11,550 cells/cm^2^). SU-DIPG-IV cells, which grow faster than SU-DIPG-VI cells, were plated in triplicates at a density of 1,000 cells per well in 96-well plates (3,300 cells/cm^2^). When specified, survival of KNS-42 was determined by plating 500 cells/well in six-well plates (55 cell/cm^2^) and performing clonogenic assay (16, 38), or by plating 60,000 cells per well in six-well plates (6,600 cells/cm^2^) for survival assay. At 24 h post-plating, cells from one triplicate were dissociated and counted to determine the cell number at the time of treatment initiation (T_0_). Seven to eight days later, the number of surviving cells was determined by counting the trypan blue-excluding cells (Te). Cell survival was expressed as a percent of control according to the following formula:


$$100\;\times \frac{AVG\;cell\;\#\;at\;Te\;(treated)-\;AVG\;cell\;\#\;at\;T0}{AVG\;cell\;\#\;at\;Te\;(control)-AVG\;cell\;\#\;at\;T0}$$

Cell death was calculated as follows:


$$100\;\times \frac{AVG\;cell\;\#\;at\;T0\;-\;AVG\;cell\;\#\;at\;Te}{AVG\;cell\;\#\;at\;T0}$$

The growth rate of HAs was higher than that of SU-DIPG-VI. Therefore, to compare the effects of the various treatments on the number of cell divisions, we plated both cell types at a density of 3,300 cells/cm^2^, which is lower than the regular density of SU-DIPG-VI (11,550 cells/cm^2^). The cells were plated in triplicates, and the number of cells at T_0_ was determined as described above. The cells were counted 7 days later (Te), and the percent of cell division relative to control was calculated according to the following formula:


$$100\;\times \frac{\log_{2}\left(AVG\;cell\;\#\;at\;Te\;(treated)/AVG\;cell\;\#\;at\;T0\right)}{\log_{2}\left(AVG\;cell\;\#\;at\;Te\;(control)/AVG\;cell\;\#\;at\;T0\right)}$$

The experimental combination indices (CIs) were obtained by employing the computer program CompuSyn, according to the method used by Chou (39). CI< 1 indicated a synergistic interaction, while CI = 1 indicated an additive interaction.

SU-DIPG-VI images were captured with the EVOS^®^ FL imaging system (Thermo Fisher Scientific, Waltham, MA) with a 10× objective.

### Radiation

The cells were irradiated in an X-ray irradiator (Polaris SC-500 series II) 24 h post-plating at a dose rate of 100 cGy/min.

### Cell lysis and Western blotting

Unless otherwise noted, treatments were initiated 24 h post-plating for DIPG cells and 48 h post-plating for KNS-42, and incubation was continued for the specified time before the cells were harvested. The cells were washed with ice-cold Dulbecco’s phosphate-buffered saline (DPBS) and collected in a buffer containing 150 mM sodium chloride (NaCl), 50 mM Tris (pH 7.5), 2% sodium dodecyl sulfate (SDS), 1.7 mM ethylenediaminetetraacetic acid, 1.5 mM sodium orthovanadate, 100 mM sodium fluoride, 1.5 μg/ml pepstatin, and Roche anti-protease cocktail. Following heating at 95°C and clearing by centrifugation, the protein concentration was determined with a bicinchoninic acid reagent (Bio-Rad, Hercules, CA). Equal loading was verified by measuring the absorbance at 520 nm of Ponceau S extracted with DPBS supplemented with 1.125 M NaCl and 0.6% Tween-20 from individual strips of a twin run (16, 38). Gels were blotted onto nitrocellulose membranes using the Trans-Blot Turbo system (Bio-Rad). Rabbit anti-P-eIF2α, anti-eIF2α, anti-eIF2Bε, and anti-HA were obtained from Cell Signaling Technology (Danvers, MA); anti-P-eIF2Bε was obtained from Thermo Fisher Scientific (Waltham, MA), anti-CD2 was obtained from Abcam (Cambridge, UK), and HRP-coupled goat anti-rabbit IgG was obtained from Jackson ImmunoResearch Laboratories (West Grove, PA). Blots were exposed to an X-ray film for chemiluminescence following treatment with West Pico ECL reagent (Thermo Fisher Scientific). Values for the integrated light density of autoradiograms were obtained with the ImageJ software and were employed to determine treatment-induced changes in protein levels.

### Transfection of cells with plasmid coding for eIF2α variants

WT, S51A, and S51D heIF2α variants in pcDNA3.CD2 expression vectors were obtained from Addgene (Cambridge, MA). WT, S51A, and S51D heIF2α in pcDNA3.1(+)-N-HA were obtained from GenScript USA Inc. (Piscataway, NJ). KNS-42 were plated in six-well plates for clonogenic assay at a density of 55 cells/cm^2^ or for survival assay at a density of 13,200 cells/cm^2^ and transfected 24 h post-plating with eIF2α variants in pcDNA3.CD2 or in pcDNA3.1(+)-N-HA by employing 0.5–1-µg/ml plasmids and jetPEI transfection reagent (Polyplus, New York, NY), according to the manufacturer’s instructions.

### Alkaline comet assay

SU-DIPG-VI and HAs cells were washed with DPBS, dissociated with diluted TrypLE™ Express Enzyme (1×) (1:4 in DPBS) (Thermo Fisher, MA), washed with cold DPBS, embedded in agar, and layered over the slides, according to the manufacturer’s instructions (Trevigen Inc., Bio-Techne Corporation, Minneapolis, MN). Following incubation with lysis buffer, the cells were further incubated in an alkali solution and then subjected to electrophoresis at 21 V for 18 min. The slides were stained with 1× SYBR Gold and viewed in a fluorescent Nikon Eclipse Ti microscope equipped with a Nikon Intensilight C-HHGFI camera using a 20× objective. The experiments were reproduced three times with DIPG and twice with HAs, and 100 cells were scored for each treatment in each experiment. Cells showing DNA tails, equal to or longer than their radii, were counted as comet-bearing cells. The images for **Figure 4** were prepared with PowerPoint software, and the same brightness (+20%) and contrast (-20%) were applied to all panels.

### Statistical analysis

The significance of the differences between the surviving fractions of the treatments relative to the untreated control cells and between the combination treatment and each of its components was verified by employing unpaired Student’s t-test. p< 0.05 in each comparison was considered statistically significant.

## Results

### The combination of vorinostat and PARPis is deleterious to PED-GBM survival

**Figure 1:**
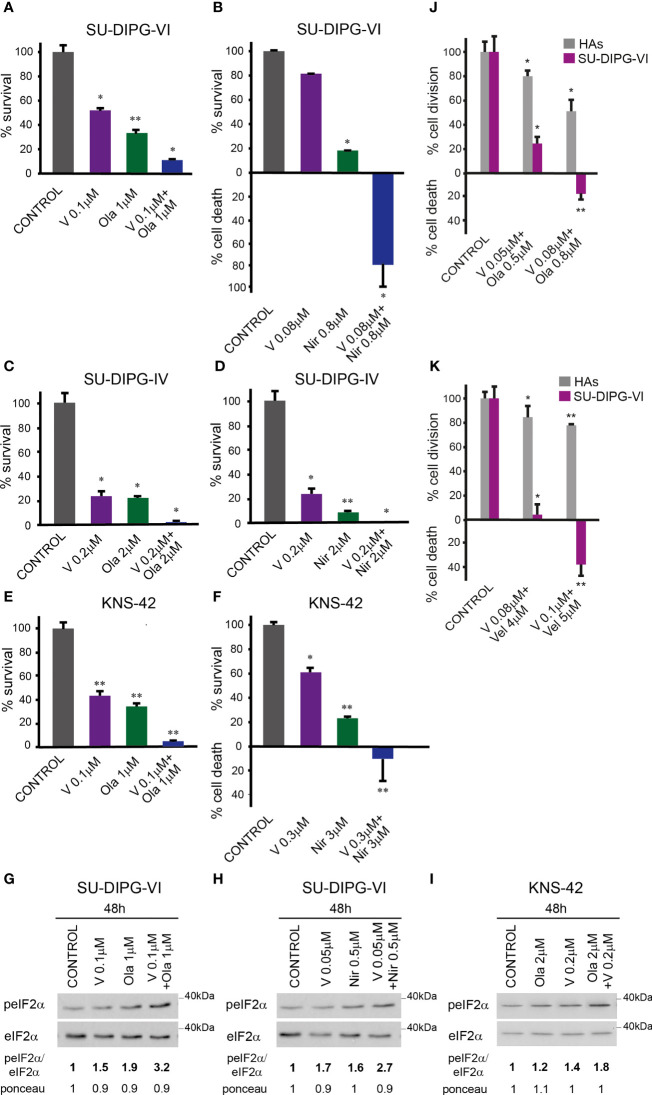
Combined treatment of vorinostat and PARPis enhanced the decrease in PED-GBM survival: SU-DIPG-VI cells **(A, B)** and SU-DIPG-IV cells **(C, D)** were plated in triplicates at a density of 11,550 or 3,300 cells/cm^2^, respectively. Drugs were added 24 h post-plating. KNS-42 were plated either for clonogenic assay **(E)** or for survival assay **(F)** at a density of 55 or 6,600 cells/cm^2^, respectively. Values are mean survival (%) ± S.D relative to control. Differences among all experimental groups, as well as between each experimental group and control, were significant - *p< 0.5, **p< 0.005. **(G–I)**. Cells were processed for Western blot analysis of treatment-induced changes in the ratio of peIF2α/eIF2α. Numbers at the bottom of the autoradiograms indicate changes of peIF2α/eIF2α and differences in loaded proteins (Ponceau) relative to control. **(J, K)**. SU-DIPG VI and HAs cells were plated in triplicates at the same density (3,300 cells/cm^2^) and treated with drugs 24 h post-plating. Values are the mean number of cell division or cell death (%) ± S.D relative to control. Differences between treated and untreated experimental groups, as well as between SU-DIPG-VI and HAs, were significant *p< 0.05, **p< 0.005. V—vorinostat, Ola—Olaparib, Vel—veliparib, Nir—niraparib. The experiments were reproduced at least twice with similar results.

To test the effect of vorinostat, PARPis, and their combination on PED-GBM survival, we employed three different specific PARPis: veliparib, olaparib, and niraparib, the most potent of which are olaparib and niraparib (40). Because the H3.3 and H3.1 K27M-mutant DIPG cell lines were reported to respond differently to treatments (41, 42), we included in our experiments both H3.3K27M-SU-DIPG-VI and H3.1K27M-SU-DIPG-IV, along with H3.3G34V-mutant KNS-42. As noted in **Figures 1A–F** and **S1**, the combination of vorinostat and PARPis decreased cell survival relative to each drug alone. Under our experimental conditions, the interaction between vorinostat and PARPis was mostly synergistic, or additive at low concentrations of niraparib and vorinostat. The SU-DIPG-VI cells were most sensitive to the combination of vorinostat and niraparib while the SU-DIPG-IV and KNS-42 cells showed similar degrees of sensitivity to the combination of vorinostat with either niraparib or olaparib. In addition, the sensitivity of PED-GBM to the combination of vorinostat and PARPis was higher than that of the HAs (**Figures 1J** **, K**). Because astrocytes grow faster in culture than PED-GBM, we had to plate for these experiments a smaller number of DIPG cells than that plated for the experiments shown in **Figures 1A, B**. This led to an observed increase in the sensitivity of PED-GBM to the drug combinations, relative to that noted in **Figures 1A, B**.

The enhanced decrease in cell survival was associated with increased P-eIF2α in the combination-treated cells relative to that triggered by each drug alone (**Figures 1G–I**). Taken together, these results suggest the possibility that increased P-eIF2α helps modulate the enhanced response to the combination of vorinostat and PARPis.

### Evaluating the role played by increased P-eIF2α in PED-GBM survival

**Figure 2:**
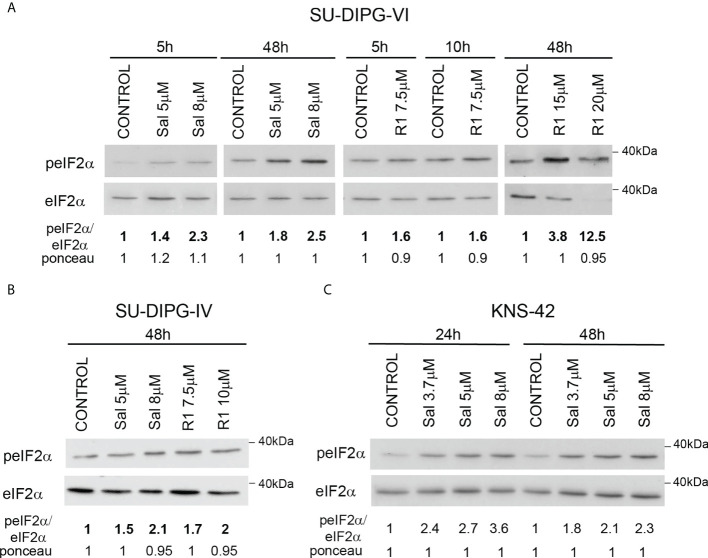
Salubrinal and raphin-1 increase P-eIF2α: **(A–C)**. Cells were processed for Western blot analysis of treatment-induced changes in the ratio of peIF2α/eIF2α at the indicated time. Numbers at the bottom of the autoradiograms indicate changes of peIF2α/eIF2α and differences in loaded proteins (Ponceau) relative to control. Sal—salubrinal, R1—raphin-1. The experiments were reproduced at least twice with similar results.

**Figure 3:**
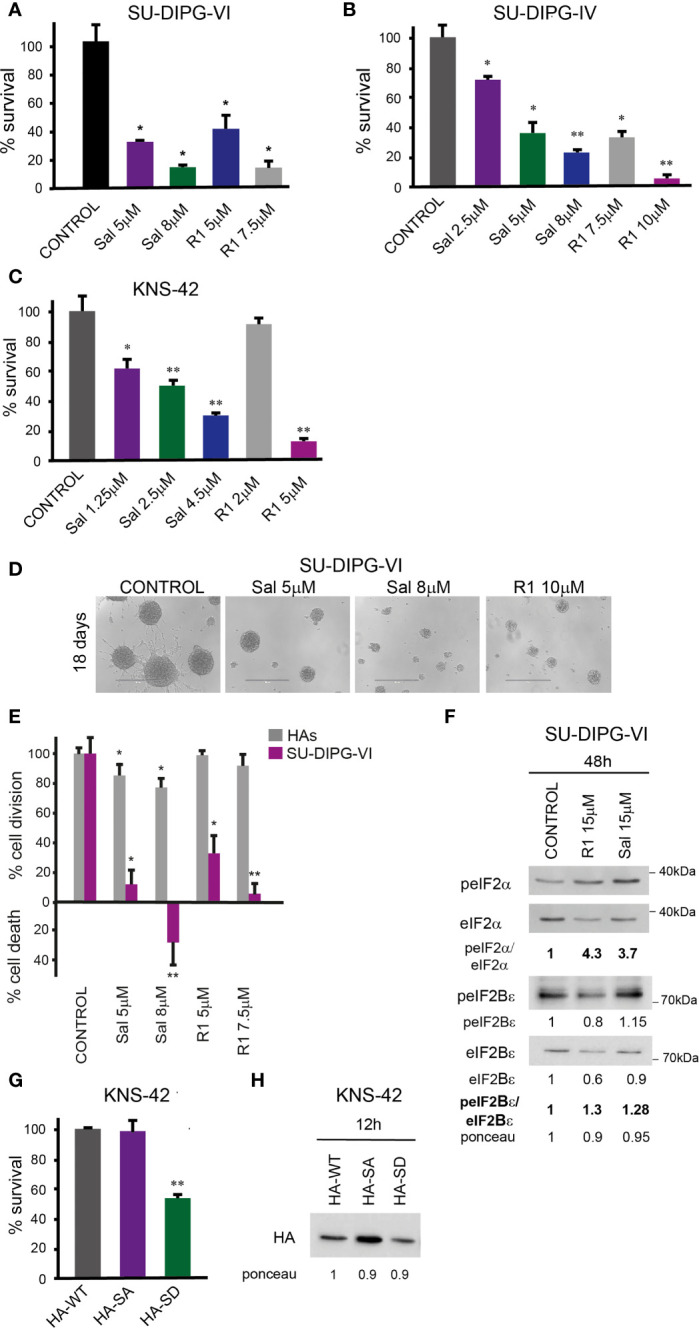
Salubrinal and raphin-1, as well as S51D-eIF2α, decreased cell survival and affected the eIF2Bε level and phosphorylation: **(A, B)**. SU-DIPG-VI and SU-DIPG-IV cells were plated in triplicates at a density of 11,550 or 3,300 cells/cm^2^, respectively, and treated 24 h post-plating. **(C)**. KNS-42 cells were plated in triplicates for clonogenic survival assay and treated 24 h post-plating. Values **(A–C)** are mean survival (%) ± S.D relative to control. Differences among all experimental groups, as well as between each experimental group and control, were significant—*p< 0.5, **p< 0.005. **(D)**. A representative image of SU-DIPG-VI neurospheres 18 days post-treatment with salubrinal and raphin-1. Bar, 400 μm. **(E)**. SU-DIPG VI and HAs cells were plated in triplicates at the same density (3,300 cells/cm^2^) and treated 24 h post-plating. Values are mean number of cell division or cell death (%) ± S.D relative to control. Differences between treated and untreated experimental groups, as well as between SU-DIPG-VI and HAs, were significant—*p< 0.05, **p< 0.005. **(F)**. SU-DIPG-VI cells were processed for Western blot analysis of treatment-induced changes in the ratios of peIF2α/eIF2α and peIF2Bε/eIF2Bε and in the cellular level of eIF2Bε. Numbers at the bottom of the autoradiograms indicate changes of peIF2α/eIF2α, peIF2Bε/eIF2Bε, and eIF2Bε and differences in loaded proteins (Ponceau) relative to control. **(G)**. KNS-42 cells were plated in duplicates at a density of 13,200 cells/cm^2^ and transiently transfected with 1 μg/ml of plasmids expressing HA-eIF2α (HA-WT) or HA-S51A-eIF2α (HA-SA) or with HA-S51D-eIF2α (HA-SD) 24 h post-plating. Values are mean survival (%) ± S.D relative to control. Differences between survival of HA-SD to the HA-WT and HA-SA were significant—**p< 0.005. **(H)**. Cells were transiently transfected as described in G and 12 h later processed for Western blot analysis of HA-tag expression. Numbers at the bottom of the autoradiograms indicate differences in loaded proteins (Ponceau) relative to HA-WT. Sal—salubrinal, R1—raphin-1. The experiments were reproduced at least twice with similar results.

**Figure 4:**
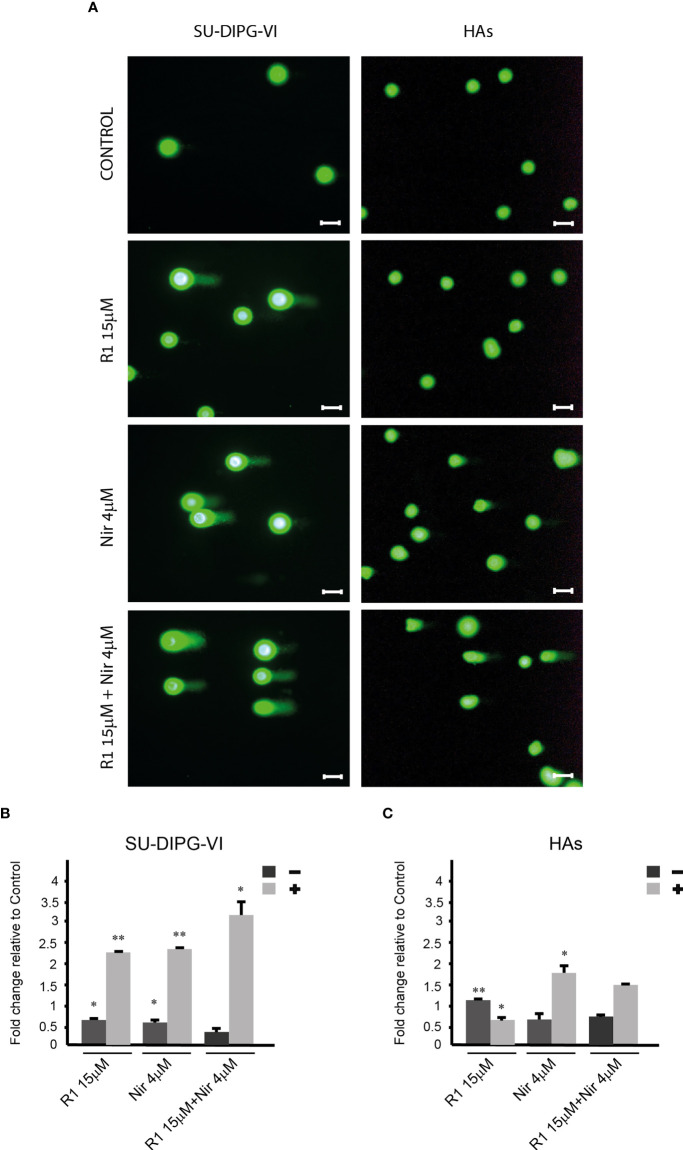
The effect of raphin-1, niraparib, and their combination on DNA integrity. SU-DIPG-VI and HAs cells were plated and treated with raphin-1, niraparib, and their combination for 48 h, before performing the comet assay. **(A)**. Images of SYBR Gold-stained cells. Bar, 100 µm. **(B, C)**. Treatment-induced changes in the fraction of cells with comets. Numbers are average ± S.D of fold change relative to control from two or three independent experiments (HAs and SU-DIPG-VI, respectively). When indicated, p-values denote significance of differences among all experimental groups, as well as between each experimental group and control—*p< 0.05, **p< 0.005. R1—Raphin-1, Nir—niraparib.

To explore the effect of increased P-eIF2α on PED-GBM survival, we employed two inhibitors of P-eIF2α dephosphorylation: salubrinal and raphin-1 (26, 30). Both drugs led to a dose-dependent increase in P-eIF2α, which was associated with decreased survival (**Figures 2** and **3A–D**). Here, PED-GBM also exhibited higher sensitivity to salubrinal or raphin-1 than HAs (**Figure 3E**). In line with these results, the comet assay showed that incubation with raphin-1 increased the fraction of cells with comet in PED-GBM but failed to do so in HAs (**Figure 4**). As mentioned earlier, eIF2B is a limiting factor in the process of regenerating the eIF2-GTP-tRNA^iMET^ ternary complex during protein translation. Therefore, even relatively small changes in its level and phosphorylation are likely to enhance the outcome of increased P-eIF2α. Unexpectedly, at 15 µM, both raphin-1 and salubrinal led to a reproducible increase in the ratio of P-eIF2Bϵ/eIF2Bϵ relative to the untreated control cells, which was associated with decreased level of eIF2Bε (**Figure 3F**, **S2** and **Table S1**). This indicates that either the eIF2Bϵ translation decreases or the molecule is being degraded, but increased kinase activity maintains a higher ratio of P-eIF2Bε/eIF2Bε. The effect of raphin-1 on the level of eIF2Bϵ was more pronounced than that of salubrinal, which may have been due to the different interactions with the hollophosphatase, as will be discussed later.

Transient transfection with the phosphomimetic S51D-eIF2α variant recapitulated the effects of salubrinal and raphin-1 on cell survival. We employed plasmids expressing N-terminal-tagged HA-eIF2α variants and plasmids expressing the eIF2α variants and a CD2 reporter (**Figures 3G** **, H** and **Figure S3**). As noted in **Figure 3G** and **S3A**, the phosphomimetic eIF2α variants expressed by either of these plasmids decreased the survival of KNS-42. Transient transfection of KNS-42 with HA-tagged eIF2α resulted in a time-dependent expression of the different variants. At 12 h post-transfection, similar expression levels of the three variants were detected with anti-HA (**Figure 3H**). At 24 and 48 h post-transfection, the expressions of HA-eIF2α and HA-S51A-eIF2α exceeded that of HA-S51D-eIF2α, which, in spite of its relatively low cellular level, markedly decreased the level of endogenous eIF2α (**Figure S3C**). This effect of the HA-tagged (S51D) variant on the level of eIF2α was similar to the effects of 15 µM salubrinal and raphin-1 (**Figures 3F**, **S2**).

### Salubrinal, raphin-1, and S51D-eIF2α increase the sensitivity of PED-GBM to PARPis

**Figure 5:**
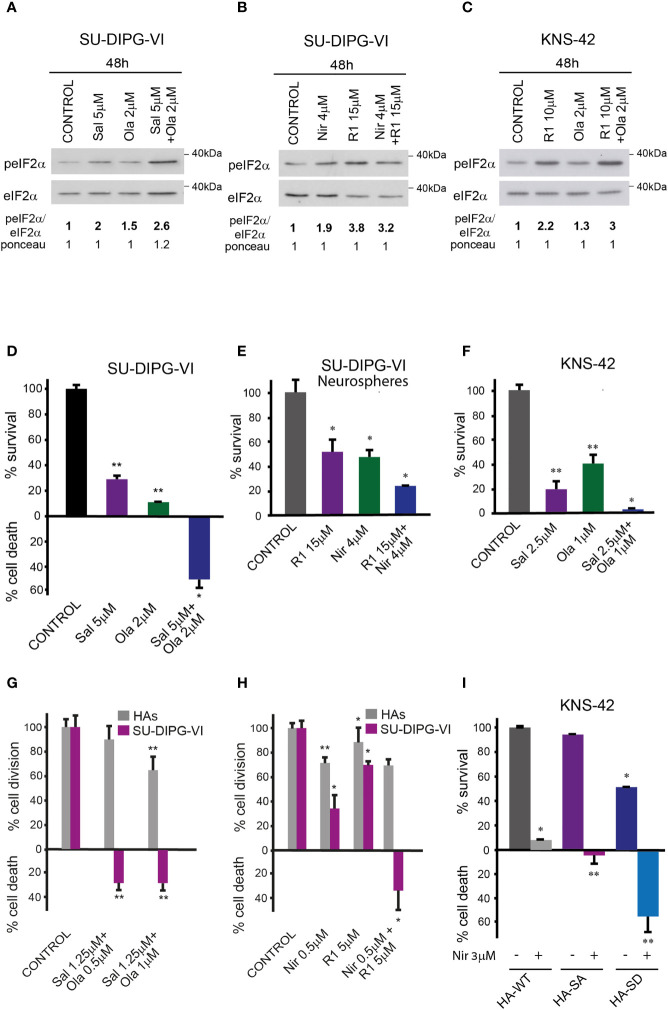
Combination of PARPis and salubrinal or raphin-1 enhanced the decrease in cell survival. **(A–C)** Cells were processed for Western blot analysis of treatment-induced changes in the ratio of peIF2α/eIF2α. Numbers at the bottom of the autoradiograms indicate changes of peIF2α/eIF2α and differences in loaded proteins (Ponceau) relative to control. **(D, E)**. SU-DIPG-VI cells were plated at a density of 11,550 cells/cm^2^, treated with drugs either 24 h **(D)** or 120 h **(E)** post-plating and incubated for additional 7 days. **(F)**. KNS-42 cells were plated in triplicates for clonogenic assay at a density of 55 cells/cm^2^ and treated with drugs 24 h post-plating. Values are mean survival or cell death (%) ± S.D relative to control. Differences among all experimental groups, as well as between each experimental group and control, were significant—*p< 0.05, **p< 0.005 **(D–F)**. **(G, H)**. SU-DIPG VI and HAs cells were plated in triplicates at a density of 3,300 cells/cm^2^ and treated with drugs 24 h post-plating. Values are mean number of cell division or cell death (%) ± S.D relative to control. Differences between treated and untreated experimental groups, as well as between SU-DIPG-VI and HAs, were significant—*p< 0.05, **p< 0.005. **(I)**. KNS-42 cells were plated in duplicates at a density of 13,200 cells/cm^2^ and transiently transfected with 1 μg/ml of plasmids expressing HA-eIF2α (HA-WT) or HA-S51A-eIF2α (HA-SA), or with HA-S51D-eIF2α (HA-SD) 24 h post-plating. Twenty-four hours later, cells were treated with niraparib for 6 days. Values are mean survival (%) ± S.D relative to control. Differences between survival of HA-SD with or without niraparib to either treated or untreated HA-SA or HA-WT were significant—*p< 0.05, **p< 0.005. Sal—salubrinal, R1—raphin-1, Ola—olaparib, Nir—niraparib. The experiments were reproduced at least twice with similar results.

The combinations of either salubrinal or raphin-1 with olaparib in SU-DIPG-VI and KNS-42 increased P-eIF2α relative to each drug alone, while the combination of raphin-1 and niraparib in SU-DIPG-VI increased P-eIF2α relative to niraparib but was similar to that obtained by raphin-1 (**Figures 5A** **–C**). Nonetheless, all the combinations enhanced the decrease in cell survival, indicating that treatment with PARPis makes the cells sensitive to increased P-eIF2α, while treatment with raphin-1 or salubrinal increases the cells’ sensitivity to PARPi-specific damage. The combination of raphin-1 and niraparib led to increased DNA damage, as manifested by the fraction of cells with comet induced by this treatment relative to each drug alone. In contrast, raphin-1 failed to increase the fraction of HAs with comet when applied alone or in combination with niraparib (**Figure 4**). As noted in **Figures 5** and **S4**, the combination of salubrinal or raphin-1 with PARPis enhanced the decrease in cell survival, whether added to single cells 48 h post-plating or to neurospheres 120 h post-plating. In addition, PED-GBM was more sensitive to the combination treatments than HAs (**Figures 5G** **, H**). Similar to salubrinal and raphin-1, the phosphomimetic S51D-eIF2α variant increased the cells’ sensitivity to niraparib relative to the non-phospohrylatable S51A or the wild-type variant (**Figure 5I**).

### Salubrinal and raphin-1 increase the sensitivity of PED-GBM to ionizing radiation

**Figure 6:**
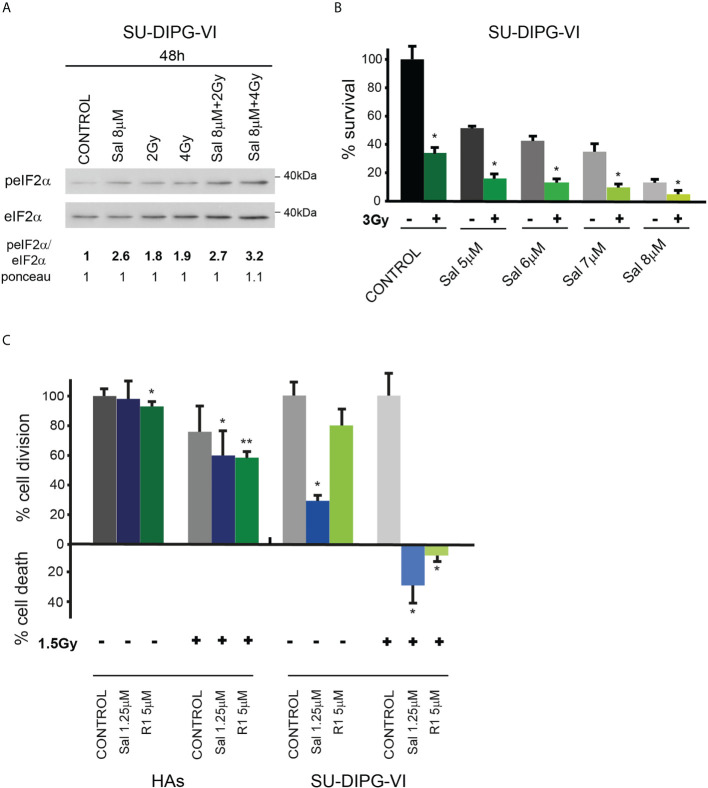
Combination of radiation with salubrinal or raphin-1 enhanced decrease in cell survival: **(A)**. SU-DIPG-VI cells were irradiated and treated with drugs 24 h post-plating and incubated for additional 48 h before processing for Western blot analysis of treatment-induced changes in the ratio of peIF2α/eIF2α. Numbers at the bottom of the autoradiograms indicate changes of peIF2α/eIF2α and differences in loaded proteins (Ponceau) relative to control. **(B)**. SU-DIPG-VI cells were plated in triplicates at a density of 11,500 cells/cm^2^ and 24 h later were irradiated and treated with salubrinal. Cells were counted 7 days following treatment initiation. Values are mean survival (%) ± S.D relative to control. Differences among all experimental groups, as well as between each experimental group and control, were significant—*p< 0.05. **(C)**. SU-DIPG VI and HA cells were plated in triplicates at a density of 3,300 cells/cm^2^ and treated with drugs and radiation 24 h post-plating. Values are mean number of cell division or cell death (%) ± S.D relative to control. Differences among all experimental groups as well as between each experimental group and control were significant for SU-DIPG-VI. When indicated, significance was observed between treated and control untreated HAs, but not among all experimental groups—*p< 0.05, **p< 0.005. Gy—gray, Sal—salubrinal, R1—Raphin-1. The experiments were reproduced once with similar results.

In light of the transient inhibitory effect of radiation on DMG tumor progression and the absence of a clinically available radio enhancer, we tested the effect of salubrinal and raphin-1 on the sensitivity of SU-DIPG-VI to ionizing radiation. As noted in **Figures 6A** **, B** and **S5**, salubrinal increased the level of P-eIF2α in the irradiated PED-GBM cells relative to that noted in the cells subjected to irradiation alone and decreased their survival relative to each treatment alone. In addition, the combination of either salubrinal or raphin-1 with 1.5 Gy, which led to cell death in DIPG cells, had only a mild effect on the proliferation of HAs (**Figure 6C**).

## Discussion

PED-GBM responded to the combination of vorinostat and PARPis by enhancing the decrease in cell survival and increasing P-eIF2α relative to that triggered by each drug alone. Tightly regulated P-eIF2α is required for maintaining metabolic demands under basal conditions and in response to stress. Thus, it has been demonstrated that a deficiency in P-eIF2α signaling due to homozygosity for eIF2α A/A and excessive phosphorylation resulting from a knockout of CReP interferes with proper embryonic development (30, 33). Therefore, to explore the role played by a sustained increase of P-eIF2α in PED-GBM survival and response to treatments, we employed inhibitors of P-eIF2α dephosphorylation and the S51D phosphomimetic variant of eIF2α.

Both salubrinal and raphin-1 led to a dose-dependent increase of P-eIF2α and decreased cell survival. Their effects on cell survival were recapitulated by phosphomimetic variants. Interestingly, the phosphomimetic variant and 15 µM salubrinal and raphin-1 decreased the level of eIF2α, which, in addition to increasing P-eIF2α, is bound to further decrease the rate of protein translation. The mechanism underlying the decreased level of eIF2α, whether by decreasing the level of its mRNA, decreasing translation, or increasing degradation, has yet to be determined.

Our results also demonstrate that 15 µM raphin-1 or salubrinal decreases the eIF2Bϵ level and increases the ratio of P-eIF2Bϵ/eIF2Bϵ, which is expected to decrease the GDP/GTP exchange activity of eIF2B. Although raphin-1 and salubrinal led to a similar increase in P-eIF2α, the effect of raphin-1 on the eIF2Bϵ level exceeded the minimal albeit reproducible effect of salubrinal. The observed difference may reflect a different interaction between the inhibitors and the hollophosphatase complex and a role played by its components other than in modulating its enzymatic activity. Thus, the interaction of raphin-1 with the hollophosphatase complex inhibits the recruitment of P-eIF2α and leads to a p97 and proteasome-dependent degradation of CReP (32). In addition to the interaction of P-eIF2α with the hollophosphatase, it interacts with CReP in complexes modulating intracellular traffic (43). Thus, a reduction in the level of CReP may increase the level of P-eIF2α, which is free to interact with eIF2B. This interaction, in contrast to ISRIB, may destabilize the eIF2B complex (44), triggering the degradation of eIF2Bϵ. Salubrinal and raphin-1 led to a similar increase of P-eIF2Bϵ/eIF2Bϵ, suggesting the decreased activity of the ERK-1/2 pathway, which is known to activate PP1 dephosphorylation and inhibition of GSK-3β, thereby suppressing P-eIF2Bϵ (19, 20).

By employing the comet assay, we demonstrated for the first time that an inhibitor of eIF2α dephosphorylation affects DNA integrity. Relevant to this finding is our previous report that demonstrated the inhibitory effect of salubrinal on the repair of DNA double-strand breaks in green fluorescent protein reporter plasmids expressed in the breast cancer cell line (16), as well as the results of Choo et al. (45) regarding the inhibitory effect of increased P-eIF2α on fork progression.

Exposing PED-GBM to the combination of PARPis with either salubrinal or raphin-1 decreased their survival relative to each drug alone. In addition, single SU-DIPG-VI cells and their neurospheres responded to the combination of raphin-1 and niraparib through an enhanced decrease in cell survival, suggesting that this combination may be effective *in vivo*. Under our experimental conditions, the level of P-eIF2α in SU-DIPG-VI treated with the combination of raphin-1 and niraparib was higher than the level of P-eIF2α in the niraparib-treated cells but was similar to that observed in the raphin-1-treated cells. This indicates that the treatment of PED-GBM with niraparib increases PED-GBM sensitivity to a further elevation of P-eIF2α, while the treatment with raphin-1 increases PED-GBM sensitivity to a specific niraparib-induced damage that is yet to be defined. Finally, similar to raphin-1 and salubrinal, the S51D phosphomimetic eIF2α variant increases the sensitivity of PED-GBM to niraparib, ascertaining the role of P-eIF2α in modulating the PED-GBM response to PARPis.

As mentioned earlier, radiation is currently the sole treatment for DMG and results in only a few months of relief. Therefore, the effects of low concentrations of raphin-1 and salubrinal on the sensitivity of PED-GBM to low doses of ionizing radiation are of immediate clinical relevance. The effects of salubrinal and raphin-1 on the brain have been reported in several studies. In mouse models, increased P-eIF2α by injecting salubrinal into the hippocampus impaired long-term memory (46). In addition, vanishing white matter syndrome is caused by the decreased activity of eIF2B (47), which can be affected by mutations or by increased P-eIF2α. In contrast, in a mouse traumatic brain injury model, salubrinal suppressed endoplasmic reticulum (ER) stress, autophagy, and apoptosis (48). Moreover, administration of sephin1, an inhibitor of GADD34, was effective in two models of neurodegenerative diseases in mice that are associated with ER stress—Charcot–Marie–Tooth 1B (CMT-1B) and SOD1-ALS (49)—and administration of raphin-1 to mouse models of Huntington disease harboring the HD^82Q^ mutation improved their weight and decreased the SDS-insoluble huntingtin and nuclear assemblies in their cortices. Importantly, unlike salubrinal, raphin-1 did not have any adverse effect on the mice’s memory (32). In addition, in contrast to its effect on PED-GBM, our experiments showed that raphin-1 decreased the fraction of cells with comet in HAs relative to the untreated control cells, and the sensitivity of HAs to raphin-1, salubrinal, and their combination with either PARPis or ionizing radiation was remarkably lower than that of PED-GBM.

PED-GBM expressing either H3.1K27M or H3.3K27M define two subgroups of DMG. They differ in the distribution of the mutated histones in their chromatin leading to differences in gene expression and dependence on metabolic pathways (41, 42, 50, 51). Also, the two variants are associated with different additional mutations. While the H3.1K27M variant is associated with mutations in ACVR1 and PI3K pathway, the H3.3K27M variant is associated with mutations in the TP53 pathway (52, 53). However, alongside these differences there are similarities—and both H3.3K27M and H3.1K27M are dependent for their survival on the wnt/β-catenin pathway (42). Defining the unique characteristics of each type of PED-GBM is extremely important for identifying molecular targets that will lead to the development of specific drugs for each type of PED-GBM, which may be more potent or specific relative to normal cells. At the moment, we do not know how and if the different H3 mutations (H3.1K27M, H3.3K27M, or H3.3G34R/V) or their associated mutations contribute to the sensitivity of PED-GBM to increased phosphorylation of eIF2α. It is possible that the different GBM will be sensitive to the same drug either because they share a common dependence on a certain pathway or because these drugs may trigger different pathways in each one of them.

In summary, our studies point to drugs that modulate P-eIF2α either alone or in combination with PARPis or ionizing radiation as novel means for treatment of PED-GBM and suggest that elucidation of their downstream effectors can reveal additional cellular components that can be targeted for the treatment of PED-GBM.

## Data availability statement

The original contributions presented in the study are included in the article/**Supplementary Material**. Further inquiries can be directed to the corresponding author.

## Author contributions

KE, SP, MY, ZV, RO, JJ-H, GR, and AT designed the study and analyzed the data. KE, SP, and MY performed the experiments. KE, SP, MY, AT, and GR wrote the paper. ML and AH revised the manuscript. All authors contributed to the article and approved its submitted version.

## Funding

This study was supported by funds from the Sheba Medical Center.

## Acknowledgments

The authors thank Dr. Michelle Monje for the generous gift of the SU-DIPG cell lines. The work was performed in partial fulfillment of the requirements for the Ph.D. degree of Ziv Versano, Sackler School of Medicine, Tel-Aviv University. Some of the data appeared in preprint in the bioRxiv server (48).

## Conflict of interest

The authors declare that the research was conducted in the absence of any commercial or financial relationships that could be construed as a potential conflict of interest.

## Publisher’s note

All claims expressed in this article are solely those of the authors and do not necessarily represent those of their affiliated organizations, or those of the publisher, the editors and the reviewers. Any product that may be evaluated in this article, or claim that may be made by its manufacturer, is not guaranteed or endorsed by the publisher.

## References

[1] Khuong-Quang DA Buczkowicz P Rakopoulos P Liu XY Fontebasso AM Bouffet E. K27M mutation in histone H3.3 defines clinically and biologically distinct subgroups of pediatric diffuse intrinsic pontine gliomas. Acta Neuropathol (2012) 124(3):439–47. doi: 10.1007/s00401-012-0998-0 PMC3422615 22661320

[2] Kallappagoudar S Yadav RK Lowe BR Partridge JF. Histone H3 mutations–a special role for H3.3 in tumorigenesis? Chromosoma (2015) 124(2):177–89. doi: 10.1007/s00412-015-0510-4 PMC4446520 25773741

[3] Harutyunyan AS Krug B Chen H Papillon-Cavanagh S Zeinieh M De Jay N. H3K27M induces defective chromatin spread of PRC2-mediated repressive H3K27me2/me3 and is essential for glioma tumorigenesis. Nat Commun (2019) 10(1):1262. doi: 10.1038/s41467-019-09140-x 30890717 PMC6425035

[4] Haag D Mack N Benites Goncalves da Silva P Statz B Clark J Tanabe K. H3.3-K27M drives neural stem cell-specific gliomagenesis in a human iPSC-derived model. Cancer Cell (2021) 39(3):407–22 e13. doi: 10.1016/j.ccell.2021.01.005 33545065

[5] Jain SU Rashoff AQ Krabbenhoft SD Hoelper D Do TJ Gibson TJ. H3 K27M and EZHIP impede H3K27-methylation spreading by inhibiting allosterically stimulated PRC2. Mol Cell (2020) 80(4):726–35 e7. doi: 10.1016/j.molcel.2020.09.028 33049227 PMC7680438

[6] Piunti A Hashizume R Morgan MA Bartom ET Horbinski CM Marshall SA. Therapeutic targeting of polycomb and BET bromodomain proteins in diffuse intrinsic pontine gliomas. Nat Med (2017) 23(4):493–500. doi: 10.1038/nm.4296 28263307 PMC5667640

[7] Nagaraja S Vitanza NA Woo PJ Taylor KR Liu F Zhang L. Transcriptional dependencies in diffuse intrinsic pontine glioma. Cancer Cell (2017) 31(5):635–52 e6. doi: 10.1016/j.ccell.2017.03.011 28434841 PMC5462626

[8] Huang TY Piunti A Qi J Morgan M Bartom E Shilatifard A. Effects of H3.3G34V mutation on genomic H3K36 and H3K27 methylation patterns in isogenic pediatric glioma cells. Acta Neuropathol Commun (2020) 8(1):219. doi: 10.1186/s40478-020-01092-4 33287886 PMC7722426

[9] Cohen KJ Pollack IF Zhou T Buxton A Holmes EJ Burger PC. Temozolomide in the treatment of high-grade gliomas in children: a report from the children's oncology group. Neuro Oncol (2011) 13(3):317–23. doi: 10.1093/neuonc/noq191 PMC3064602 21339192

[10] Grasso CS Tang Y Truffaux N Berlow NE Liu L Debily MA. Functionally defined therapeutic targets in diffuse intrinsic pontine glioma. Nat Med (2015) 21(7):827. doi: 10.1038/nm.3855 26151328

[11] Chornenkyy Y Agnihotri S Yu M Buczkowicz P Rakopoulos P Golbourn B. Poly-ADP-Ribose polymerase as a therapeutic target in pediatric diffuse intrinsic pontine glioma and pediatric high-grade astrocytoma. Mol Cancer Ther (2015) 14(11):2560–8. doi: 10.1158/1535-7163.MCT-15-0282 26351319

[12] Yalon M Tuval-Kochen L Castel D Moshe I Mazal I Cohen O. Overcoming resistance of cancer cells to PARP-1 inhibitors with three different drug combinations. PloS One (2016) 11(5):e0155711. doi: 10.1371/journal.pone.0155711 27196668 PMC4873128

[13] Chao OS Goodman OB Jr. Synergistic loss of prostate cancer cell viability by coinhibition of HDAC and PARP. Mol Cancer Res (2014) 12(12):1755–66. doi: 10.1158/1541-7786.MCR-14-0173 25127709

[14] Gaymes TJ Shall S MacPherson LJ Twine NA Lea NC Farzaneh F. Inhibitors of poly ADP-ribose polymerase (PARP) induce apoptosis of myeloid leukemic cells: potential for therapy of myeloid leukemia and myelodysplastic syndromes. Haematologica (2009) 94(5):638–46. doi: 10.3324/haematol.2008.001933 PMC2675675 19407318

[15] Konstantinopoulos PA Wilson AJ Saskowski J Wass E Khabele D. Suberoylanilide hydroxamic acid (SAHA) enhances olaparib activity by targeting homologous recombination DNA repair in ovarian cancer. Gynecol Oncol (2014) 133(3):599–606. doi: 10.1016/j.ygyno.2014.03.007 24631446 PMC4347923

[16] Tuval-Kochen L Paglin S Keshet G Lerenthal Y Nakar C Golani T. Eukaryotic initiation factor 2alpha–a downstream effector of mammalian target of rapamycin–modulates DNA repair and cancer response to treatment. PloS One (2013) 8(10):e77260. doi: 10.1371/journal.pone.0077260 24204783 PMC3808413

[17] Versano Z Shany E Freedman S Tuval-Kochen L Leitner M Paglin S. MutT homolog 1 counteracts the effect of anti-neoplastic treatments in adult and pediatric glioblastoma cells. Oncotarget (2018) 9(44):27547–63. doi: 10.18632/oncotarget.25547 PMC6007941 29938005

[18] Wek RC. Role of eIF2alpha kinases in translational control and adaptation to cellular stress. Cold Spring Harb Perspect Biol (2018) 10(7). doi: 10.1101/cshperspect.a032870 PMC6028073 29440070

[19] Cagnetta R Wong HH Frese CK Mallucci GR Krijgsveld J Holt CE. Noncanonical modulation of the eIF2 pathway controls an increase in local translation during neural wiring. Mol Cell (2019) 73(3):474–89 e5. doi: 10.1016/j.molcel.2018.11.013 30595434 PMC6375727

[20] Quevedo C Salinas M Alcazar A. Initiation factor 2B activity is regulated by protein phosphatase 1, which is activated by the mitogen-activated protein kinase-dependent pathway in insulin-like growth factor 1-stimulated neuronal cells. J Biol Chem (2003) 278(19):16579–86. doi: 10.1074/jbc.M212936200 12624094

[21] Baird TD Wek RC. Eukaryotic initiation factor 2 phosphorylation and translational control in metabolism. Adv Nutr (2012) 3(3):307–21. doi: 10.3945/an.112.002113 PMC3649462 22585904

[22] Novoa I Zeng H Harding HP Ron D. Feedback inhibition of the unfolded protein response by GADD34-mediated dephosphorylation of eIF2alpha. J Cell Biol (2001) 153(5):1011–22. doi: 10.1083/jcb.153.5.1011 PMC2174339 11381086

[23] Harding HP Zhang Y Scheuner D Chen JJ Kaufman RJ Ron D. Ppp1r15 gene knockout reveals an essential role for translation initiation factor 2 alpha (eIF2alpha) dephosphorylation in mammalian development. Proc Natl Acad Sci U.S.A. (2009) 106(6):1832–7. doi: 10.1073/pnas.0809632106 PMC2644123 19181853

[24] Tian X Zhang S Zhou L Seyhan AA Hernandez Borrero L Zhang Y. Targeting the integrated stress response in cancer therapy. Front Pharmacol (2021) 12:747837. doi: 10.3389/fphar.2021.747837 34630117 PMC8498116

[25] Jousse C Oyadomari S Novoa I Lu P Zhang Y Harding HP. Inhibition of a constitutive translation initiation factor 2alpha phosphatase, CReP, promotes survival of stressed cells. J Cell Biol (2003) 163(4):767–75. doi: 10.1083/jcb.200308075 PMC2173671 14638860

[26] Chen R Rato C Yan Y Crespillo-Casado A Clarke HJ Harding HP. G-Actin provides substrate-specificity to eukaryotic initiation factor 2alpha holophosphatases. Elife (2015) 4. doi: 10.7554/eLife.04871 PMC4394352 25774600

[27] Chambers JE Dalton LE Clarke HJ Malzer E Dominicus CS Patel V. Actin dynamics tune the integrated stress response by regulating eukaryotic initiation factor 2alpha dephosphorylation. Elife (2015) 4. doi: 10.7554/eLife.04872 PMC4394351 25774599

[28] Boyce M Bryant KF Jousse C Long K Harding HP Scheuner D. A selective inhibitor of eIF2alpha dephosphorylation protects cells from ER stress. Science (2005) 307(5711):935–9. doi: 10.1126/science.1101902 15705855

[29] Cnop M Ladriere L Hekerman P Ortis F Cardozo AK Dogusan Z. Selective inhibition of eukaryotic translation initiation factor 2 alpha dephosphorylation potentiates fatty acid-induced endoplasmic reticulum stress and causes pancreatic beta-cell dysfunction and apoptosis. J Biol Chem (2007) 282(6):3989–97. doi: 10.1074/jbc.M607627200 17158450

[30] Scheuner D Song B McEwen E Liu C Laybutt R Gillespie P. Translational control is required for the unfolded protein response and *in vivo* glucose homeostasis. Mol Cell (2001) 7(6):1165–76. doi: 10.1016/S1097-2765(01)00265-9 11430820

[31] Crespillo-Casado A Chambers JE Fischer PM Marciniak SJ Ron D. PPP1R15A-mediated dephosphorylation of eIF2alpha is unaffected by Sephin1 or guanabenz. Elife (2017) 6. doi: 10.7554/eLife.26109 PMC5429092 28447936

[32] Krzyzosiak A Sigurdardottir A Luh L Carrara M Das I Schneider K. Target-based discovery of an inhibitor of the regulatory phosphatase PPP1R15B. Cell (2018) 174(5):1216–28 e19. doi: 10.1016/j.cell.2018.06.030 30057111 PMC6108835

[33] Schoof M Boone M Wang L Lawrence R Frost A Walter P. eIF2B conformation and assembly state regulate the integrated stress response. Elife (2021) 10. doi: 10.7554/eLife.65703.sa2 PMC7990499 33688831

[34] Peidis P Papadakis AI Muaddi H Richard S Koromilas AE. Doxorubicin bypasses the cytoprotective effects of eIF2alpha phosphorylation and promotes PKR-mediated cell death. Cell Death Differ (2011) 18(1):145–54. doi: 10.1038/cdd.2010.76 PMC3131862 20559319

[35] Persaud L Zhong X Alvarado G Do W Dejoie J Zybtseva A. eIF2alpha phosphorylation mediates IL24-induced apoptosis through inhibition of translation. Mol Cancer Res (2017) 15(8):1117–24. doi: 10.1158/1541-7786.MCR-16-0454 PMC5743333 28461326

[36] Chi AS Tarapore RS Hall MD Shonka N Gardner S Umemura Y. Pediatric and adult H3 K27M-mutant diffuse midline glioma treated with the selective DRD2 antagonist ONC201. J Neurooncol (2019) 145(1):97–105. doi: 10.1007/s11060-019-03271-3 31456142 PMC7241441

[37] Kline CL Van den Heuvel AP Allen JE Prabhu VV Dicker DT El-Deiry WS. ONC201 kills solid tumor cells by triggering an integrated stress response dependent on ATF4 activation by specific eIF2alpha kinases. Sci Signal (2016) 9(415):ra18. doi: 10.1126/scisignal.aac4374 26884600 PMC4968406

[38] Paglin S Lee NY Nakar C Fitzgerald M Plotkin J Deuel B. Rapamycin-sensitive pathway regulates mitochondrial membrane potential, autophagy, and survival in irradiated MCF-7 cells. Cancer Res (2005) 65(23):11061–70. doi: 10.1158/0008-5472.CAN-05-1083 16322256

[39] Chou TC. Drug combination studies and their synergy quantification using the chou-talalay method. Cancer Res (2010) 70(2):440–6. doi: 10.1158/0008-5472.CAN-09-1947 20068163

[40] Min A Im SA. PARP inhibitors as therapeutics: Beyond modulation of PARylation. Cancers (Basel) (2020) 12(2):394. doi: 10.3390/cancers12020394 PMC7072193 32046300

[41] Castel D Philippe C Calmon R Le Dret L Truffaux N Boddaert N. Histone H3F3A and HIST1H3B K27M mutations define two subgroups of diffuse intrinsic pontine gliomas with different prognosis and phenotypes. Acta Neuropathol (2015) 130(6):815–27. doi: 10.1007/s00401-015-1478-0 PMC4654747 26399631

[42] Nagaraja S Quezada MA Gillespie SM Arzt M Lennon JJ Woo PJ. Histone variant and cell context determine H3K27M reprogramming of the enhancer landscape and oncogenic state. Mol Cell (2019) 76(6):965–80 e12. doi: 10.1016/j.molcel.2019.08.030 31588023 PMC7104854

[43] Kloft N Neukirch C von Hoven G Bobkiewicz W Weis S Boller K. A subunit of eukaryotic translation initiation factor 2alpha-phosphatase (CreP/PPP1R15B) regulates membrane traffic. J Biol Chem (2012) 287(42):35299–317. doi: 10.1074/jbc.M112.379883 PMC3471756 22915583

[44] Bogorad AM Lin KY Marintchev A. Novel mechanisms of eIF2B action and regulation by eIF2alpha phosphorylation. Nucleic Acids Res (2017) 45(20):11962–79. doi: 10.1093/nar/gkx845 PMC5714165 29036434

[45] Choo J Schlosser D Manzini V Magerhans A Dobbelstein M. The integrated stress response induces r-loops and hinders replication fork progression. Cell Death Dis (2020) 11(7):538. doi: 10.1038/s41419-020-2727-2 32678076 PMC7366693

[46] Costa-Mattioli M Gobert D Stern E Gamache K Colina R Cuello C. eIF2alpha phosphorylation bidirectionally regulates the switch from short- to long-term synaptic plasticity and memory. Cell (2007) 129(1):195–206. doi: 10.1016/j.cell.2007.01.050 17418795 PMC4149214

[47] Wong YL LeBon L Basso AM Kohlhaas KL Nikkel AL Robb HM. eIF2B activator prevents neurological defects caused by a chronic integrated stress response. Elife (2019) 8. doi: 10.7554/eLife.42940 PMC6326728 30624206

[48] Wang ZF Gao C Chen W Gao Y Wang HC Meng Y. Salubrinal offers neuroprotection through suppressing endoplasmic reticulum stress, autophagy and apoptosis in a mouse traumatic brain injury model. Neurobiol Learn Mem (2019) 161:12–25. doi: 10.1016/j.nlm.2019.03.002 30851432

[49] Das I Krzyzosiak A Schneider K Wrabetz L D'Antonio M Barry N. Preventing proteostasis diseases by selective inhibition of a phosphatase regulatory subunit. Science (2015) 348(6231):239–42. doi: 10.1126/science.aaa4484 PMC4490275 25859045

[50] Sarthy JF Meers MP Janssens DH Henikoff JG Feldman H Paddison PJ. Histone deposition pathways determine the chromatin landscapes of H3.1 and H3.3 K27M oncohistones. Elife (2020) 9. doi: 10.7554/eLife.61090 PMC7518889 32902381

[51] Sturm D Witt H Hovestadt V Khuong-Quang DA Jones DT Konermann C. Hotspot mutations in H3F3A and IDH1 define distinct epigenetic and biological subgroups of glioblastoma. Cancer Cell (2012) 22(4):425–37. doi: 10.1016/j.ccr.2012.08.024 23079654

[52] Buczkowicz P Hoeman C Rakopoulos P Pajovic S Letourneau L Dzamba M. Genomic analysis of diffuse intrinsic pontine gliomas identifies three molecular subgroups and recurrent activating ACVR1 mutations. Nat Genet (2014) 46(5):451–6. doi: 10.1038/ng.2936 PMC3997489 24705254

[53] Fontebasso AM Papillon-Cavanagh S Schwartzentruber J Nikbakht H Gerges N Fiset PO. Recurrent somatic mutations in ACVR1 in pediatric midline high-grade astrocytoma. Nat Genet (2014) 46(5):462–6. doi: 10.1038/ng.2950 PMC4282994 24705250

