# Supplementary material for: Pediatric glioblastoma cells are sensitive to drugs that inhibit eIF2α dephosphorylation and its phosphomimetic S51D variant
Source: Front Oncol. 2022 Aug 26;12:959133. doi: 10.3389/fonc.2022.959133 (PMC9462064; doi:10.3389/fonc.2022.959133)
Supplement: Supplementary file 1 [file DataSheet_1.pdf]

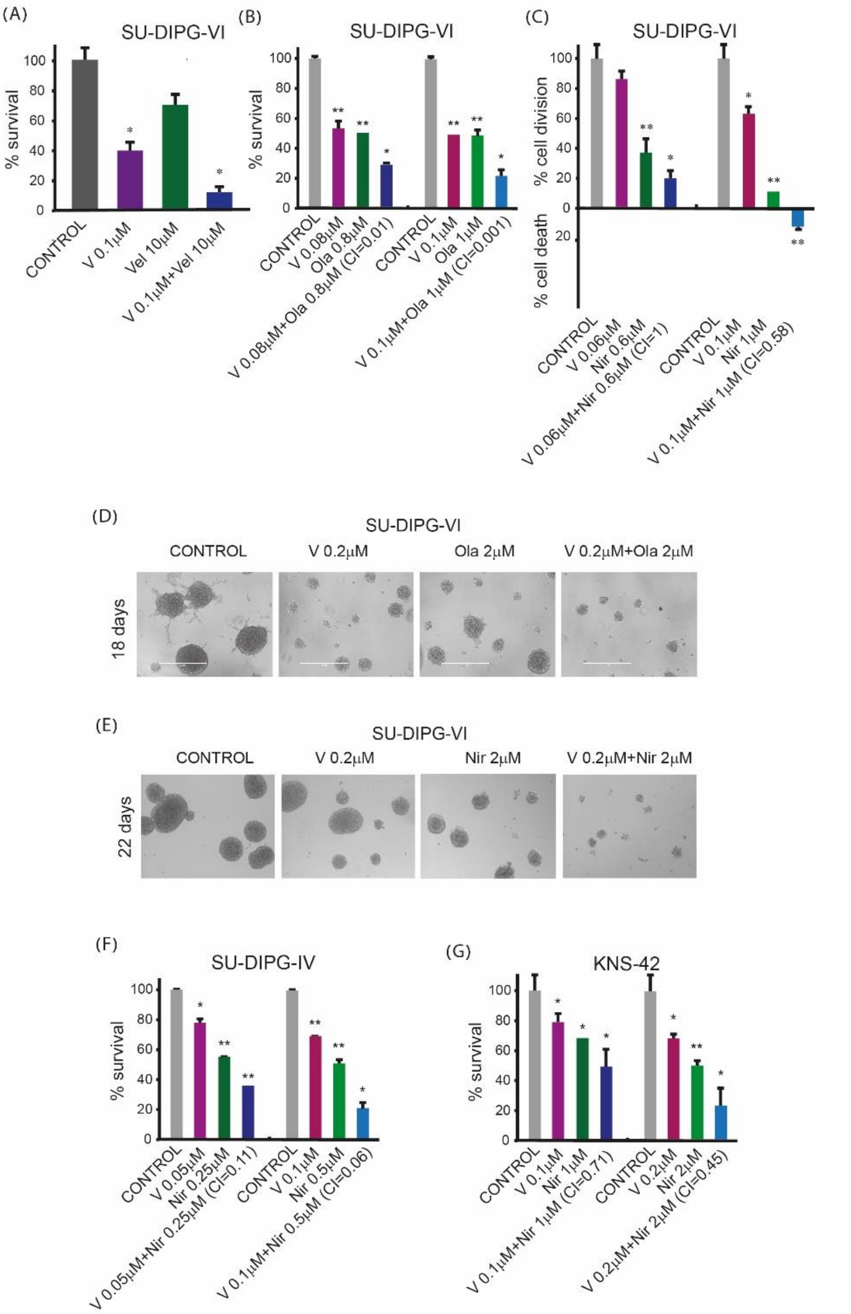

Supplementary Figure 1: **Combined treatment of vorinostat and PARPis enhanced decrease in PED-GBM survival**: (**A-C**)**.** SU-DIPG-VI cells were plated in triplicates at a density of 11500 cells/cm^2 and treated with drugs 24 h post-plating. Values are mean survival or cell death (%) ± S.D relative to control. Differences among all experimental groups, as well as between each experimental group and control, were significant - *$p<0.05$, **$p<0.005$. (**D,E**). Representative images of SU-DIPG-VI neurospheres 18-22 days post- treatment with vorinostat 0.2μM, olaparib 2μM and their combination (D) or with vorinostat 0.2μM and niraparib 2μM and their combination (E). Bar, 400μm. (F,G). SU-DIPG-IV and KNS-42 were plated at a density of 3300 and 6600 cells/cm^2 respectively and treated 24 h post-plating. Values are mean survival or cell death (%) ± S.D relative to control. Differences among all experimental groups, as well as between each experimental group and control, were significant - *$p<0.05$, **$p<0.005$.

V-vorinostat, Vel – veliparib, Ola- olaparib, Nir- niraparib, CI- combination index.

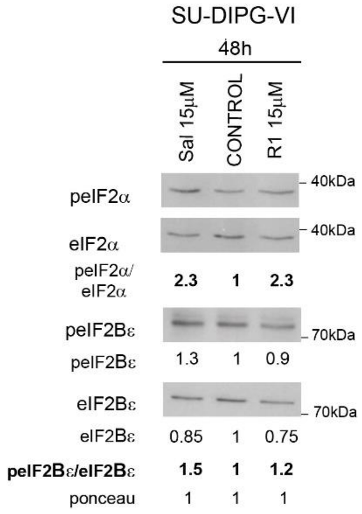

Supplementary Figure 2: **Salubrinal and raphin-1 affected eIF2Bε level and phosphorylation**: SU-DIPG-VI cells were processed for western blot analysis of treatment-induced changes in the ratio of peIF2α/eIF2α, peIF2Bε/eIF2Bε and in the cellular level of eIF2Bε as described in methods. Numbers at the bottom of the autoradiograms indicate changes of peIF2α/eIF2α, peIF2Bε/eIF2Bε, eIF2Bε and differences in loaded proteins (Ponceau) relative to control. Sal- salubrinal, R1 – raphin-1.

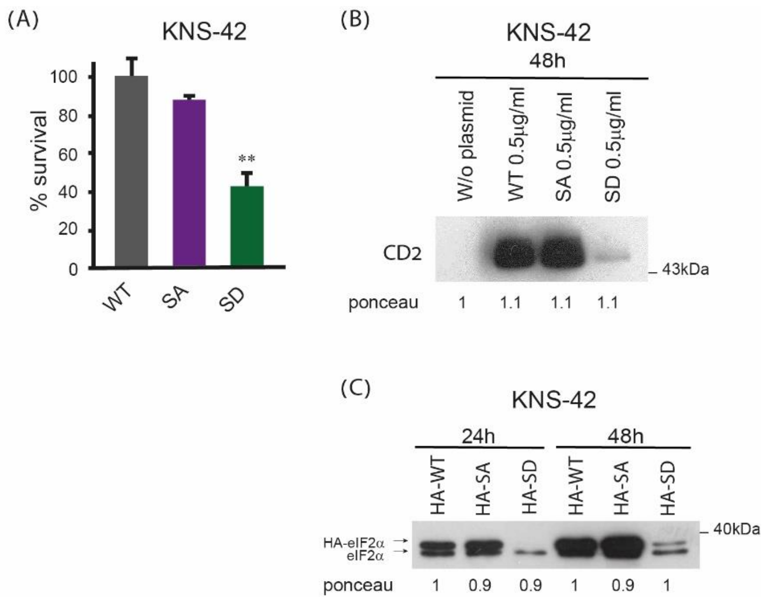

Supplementary Figure 3: **Phosphomimetic eIF2α variants decrease survival of KNS-42:** (**A,B**). KNS-42 cells were plated in duplicates and 24 h post-plating were transiently transfected with 0.5µg/ml plasmids expressing eIF2α variants, as well as CD2. (A)**.** Values are mean survival (%) ± S.D relative to control. Differences between survival of S51D-eIF2α (SD) to the eIF2α (WT) and S51A-eIF2α (SA) were significant- **p<0.005. (B)**.** Transfected KNS-42 cells were processed for western blot analysis of CD2 expression. Numbers at the bottom of the autoradiograms differences in loaded proteins (Ponceau). (**C**)**.** KNS-42 cells were transiently transfected with 1µg/ml of plasmids expressing HA-tagged eIF2α variants, as described in methods. The cells where than processed for western blot analysis of expression of endogenous and HA-tagged eIF2α variants. Numbers at the bottom of the autoradiograms indicate differences of loaded proteins (Ponceau).

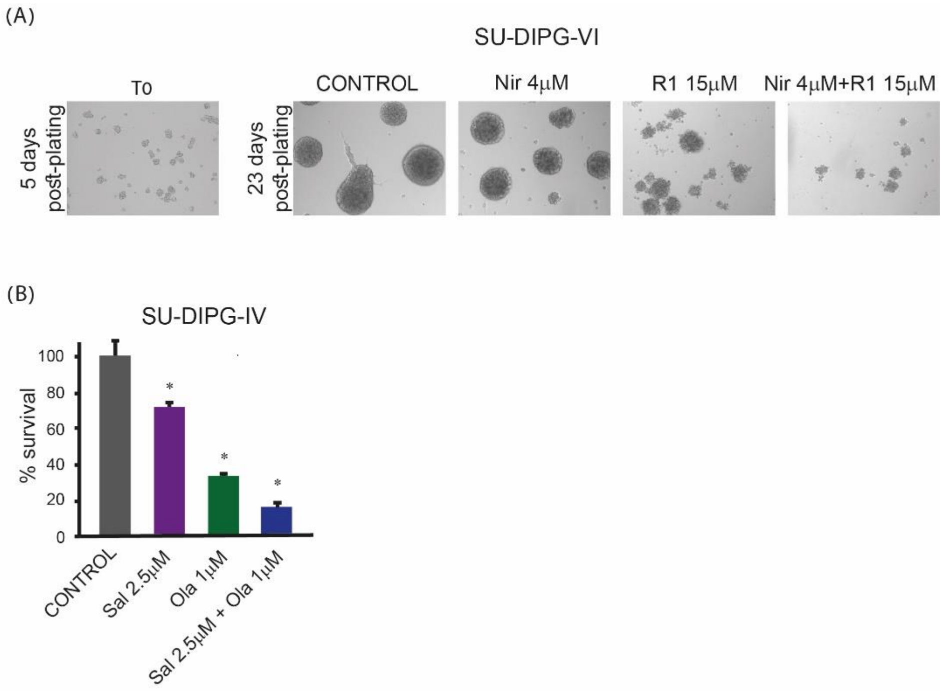

Supplementary Figure 4: **Combination of niraparib and raphin-1 enhanced decrease in survival of neurospheres**. (A). Cells were treated 120 h (T0) post-plating and images were captured 18 days post-treatment initiation with 4μM niraparib and 15μM raphin-1. Bar, 400μm. (B). SU-DIPG-IV cells were plated in triplicates at a density of 3300 cells/cm^2 and treated with drugs 24 h post-plating. Values are mean survival (%) ± S.D relative to control. Differences among all experimental groups, as well as between each experimental group and control, were significant - *$p<0.05$. Nir – niraparib, R1 – raphin-1, Sal – salubrinal, Ola – Olaparib.

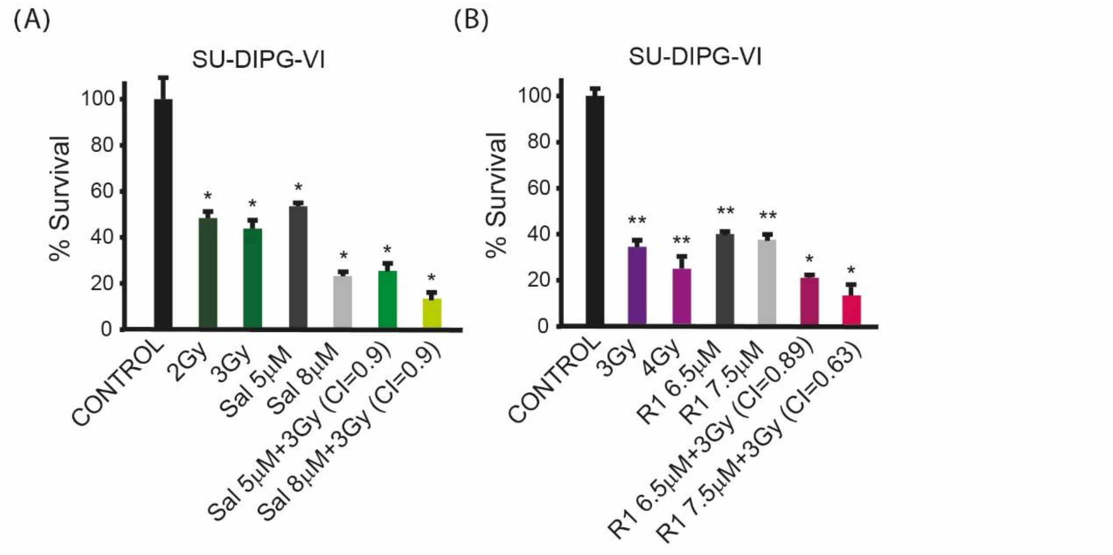

Supplementary Figure 5: C**ombination of radiation with salubrinal or raphin-1 enhanced decrease in cell survival:** SU-DIPG-VI cells were plated in triplicates at a density of 11500 cells/cm^2 and 24 h later were irradiated and treated with salubrinal (A) or raphin-1 (B). Cells were counted 7 days following treatment initiation. Values are mean survival (%) ± S.D relative to control. Differences among all experimental groups, as well as between each experimental group and control, were significant - *p<0.05, **p<0.005. Gy-gray, Sal - salubrinal, R1- raphin-1, CI - combination index.
