# Supplementary material for: Pediatric glioblastoma cells are sensitive to drugs that inhibit eIF2α dephosphorylation and its phosphomimetic S51D variant
Source: Front Oncol. 2022 Aug 26;12:959133. doi: 10.3389/fonc.2022.959133 (PMC9462064; doi:10.3389/fonc.2022.959133)
Supplement: Supplementary file 2 [file Table_1.docx]

**Supplementary Table 1**: **Salubrinal and raphin-1 affected eIF2Bε level and phosphorylation**: SU-DIPG-VI cells were processed for western blot analysis of treatment-induced changes in the ratio of P-eIF2Bε/eIF2Bε and in the cellular levels of P-eIF2Bε and eIF2Bε as described in Fig. 3F and its legend. Quantification of treatment-induce changes was performed with ImageJ software.

| **exp. #** | **treatment** | **peIF2Bε** | **eIF2Bε** | **peIF2Bε/eIF2Bε** |
| --- | --- | --- | --- | --- |
| 1 | R1 15μM | 1.2 | 0.6 | 2 |
| 2 | R1 15μM | 0.9 | 0.75 | 1.2 |
| 3 | R1 15μM | 0.8 | 0.6 | 1.33 |
| 4 | R1 15μM | N.D | 0.7 | N.D |
| 5 | Sal 15μM | 1.3 | 0.85 | 1.53 |
| 6 | Sal 15μM | 1.15 | 0.9 | 1.28 |
